# Genomic landscape of mature B-cell non-Hodgkin lymphomas — an appraisal from lymphomagenesis to drug resistance

**DOI:** 10.1186/s43046-022-00154-z

**Published:** 2022-12-12

**Authors:** Devasis Panda, Nupur Das, Deepshi Thakral, Ritu Gupta

**Affiliations:** Department of Laboratory Oncology, Dr. BRAIRCH, AIIMS, New Delhi, 110029 India

**Keywords:** B-cell non-Hodgkin lymphoma, Genomics, Molecular alterations, Lymphomagenesis

## Abstract

**Background:**

Mature B-cell non-Hodgkin lymphomas are one of the most common hematological malignancies with a divergent clinical presentation, phenotype, and course of disease regulated by underlying genetic mechanism.

**Main body:**

Genetic and molecular alterations are not only critical for lymphomagenesis but also largely responsible for differing therapeutic response in these neoplasms. In recent years, advanced molecular tools have provided a deeper understanding regarding these oncogenic drives for predicting progression as well as refractory behavior in these diseases. The prognostic models based on gene expression profiling have also been proved effective in various clinical scenarios. However, considerable overlap does exist between the genotypes of individual lymphomas and at the same time where additional molecular lesions may be associated with each entity apart from the key genetic event. Therefore, genomics is one of the cornerstones in the multimodality approach essential for classification and risk stratification of B-cell non-Hodgkin lymphomas.

**Conclusion:**

We hereby in this review discuss the wide range of genetic aberrancies associated with tumorigenesis, immune escape, and chemoresistance in major B-cell non-Hodgkin lymphomas.

## Background

Recent years have witnessed a significant advancement in the diagnostic modalities of mature B-cell neoplasms guided towards targeted therapeutic intervention leading to superior prognostication. This vast and divergent group of clonal malignancies requires a multifaceted approach including morphology, immunohistochemistry (IHC), flow cytometry, cytogenetics, and molecular studies for an accurate and pinpoint diagnosis. Simultaneously, correlation with clinical features and presentation is critical as these neoplasms manifest varied disease courses ranging from indolent to highly aggressive tumors [1]. Molecular biology in B-cell non-Hodgkin lymphomas (B-NHLs) too is quite heterogeneous and has a consequential impact on the phenotype as well as outcome. A broad spectrum of alterations in genetic profile including aneuploidies, structural rearrangement of chromosomes, copy number variations, and point mutations have been described in these lymphomas [1, 2].

Identification of hallmark genetic aberrancies through interphase fluorescence in situ hybridization (FISH) is one of the important tools for the diagnosis of certain lymphomas notably mantle cell lymphomas (MCLs) with *t (11;14) (q13;q32)*, follicular lymphomas (FLs) with *t(14;18) (q32;q21)*, Burkitt lymphomas (BLs) with *t(8;14) (q24;q32)*, and high-grade B-cell lymphoma (HGBLs) with *MYC*, *BCL2*, and/or *BCL6* rearrangements (double/triple-hit lymphomas) [1, 3–5]. Few recent clinical prognostic models have also proposed specific FISH characteristics in diffuse large B-cell lymphomas (DLBCLs) [6, 7].

Nonetheless, the advent of advanced molecular techniques like targeted sequencing, RNA transcript quantification, cell-free DNA techniques, and multiplex ligation-dependent probe amplification (MLPA)-based assays continues to refine the genotype and risk stratification of individual disease [8, 9]. With the advent of novel mutations such as *BRAF V600E* in hairy cell leukemia (HCL) and *MYD88 L265P* in lymphoplasmacytic lymphoma (LPL), the interpretation of these entities has become a lot easier [10, 11]. Furthermore, the additional genetic mechanism such as missense and truncating mutations of chromatin modifiers like *CREBBP*, *EZH2*, and *DDX3X* as well as histone methylation such as *KMT2D* and *SUZ12* in DLBCLs has given immense insight into the role of epigenetics in B-NHL pathogenesis [8, 12, 13].

The genetic alterations lead to dysregulation within the common intracellular pathways involved in ontogeny and maturation of B lymphocyte such as *B-cell antigen receptor* (*BCR*), *nuclear factor kappa B* (*NF-κB*), and *PI3K/AKT/mTOR* signaling pathways [14]. It is noteworthy that automation as well as advanced technological tools in diagnostic genomics has revealed the complex biology of B-NHL. These cytogenetic and molecular techniques are now being routinely used on paraffin-embedded biopsies, frozen tumor sections, fine needle aspirate samples, and liquid hematological specimens like peripheral blood and bone marrow aspirate. Herein, we attempt to review the wide range of genetic aberrancies in individual B-NHLs and their key role in lymphomagenesis.

## Overview of B-NHL diagnosis and classification

Mature B-cell neoplasms constitute approximately > 90% of lymphoid malignancies worldwide with a broad age distribution. The classification of B-NHLs is essentially based on the following: (1) ontogenic classification — based on B-cell ontogeny and differentiation; (2) morphological classification — based on B-cell size and morphology; (3) immunophenotypic classification — based on expression of various antigens; and (4) clinical classification — based on clinical behaviour, aggressiveness, and overall outcome.

**Fig. 1:**
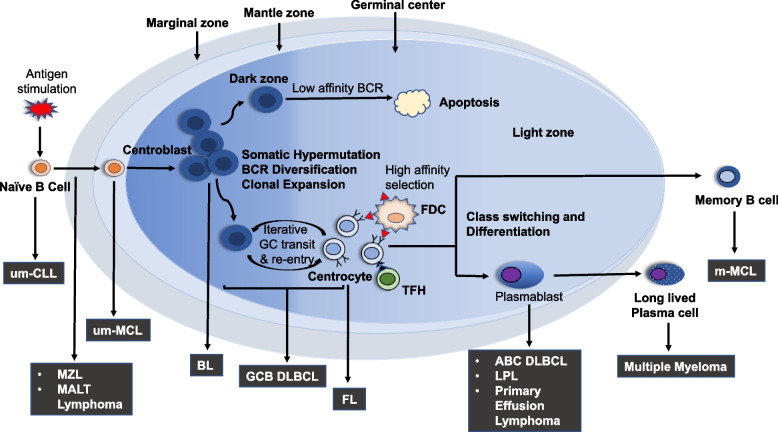
B-cell ontogeny and differentiation in the lymph node germinal center and its major neoplastic equivalents during lymphomagenesis

**Fig. 2:**
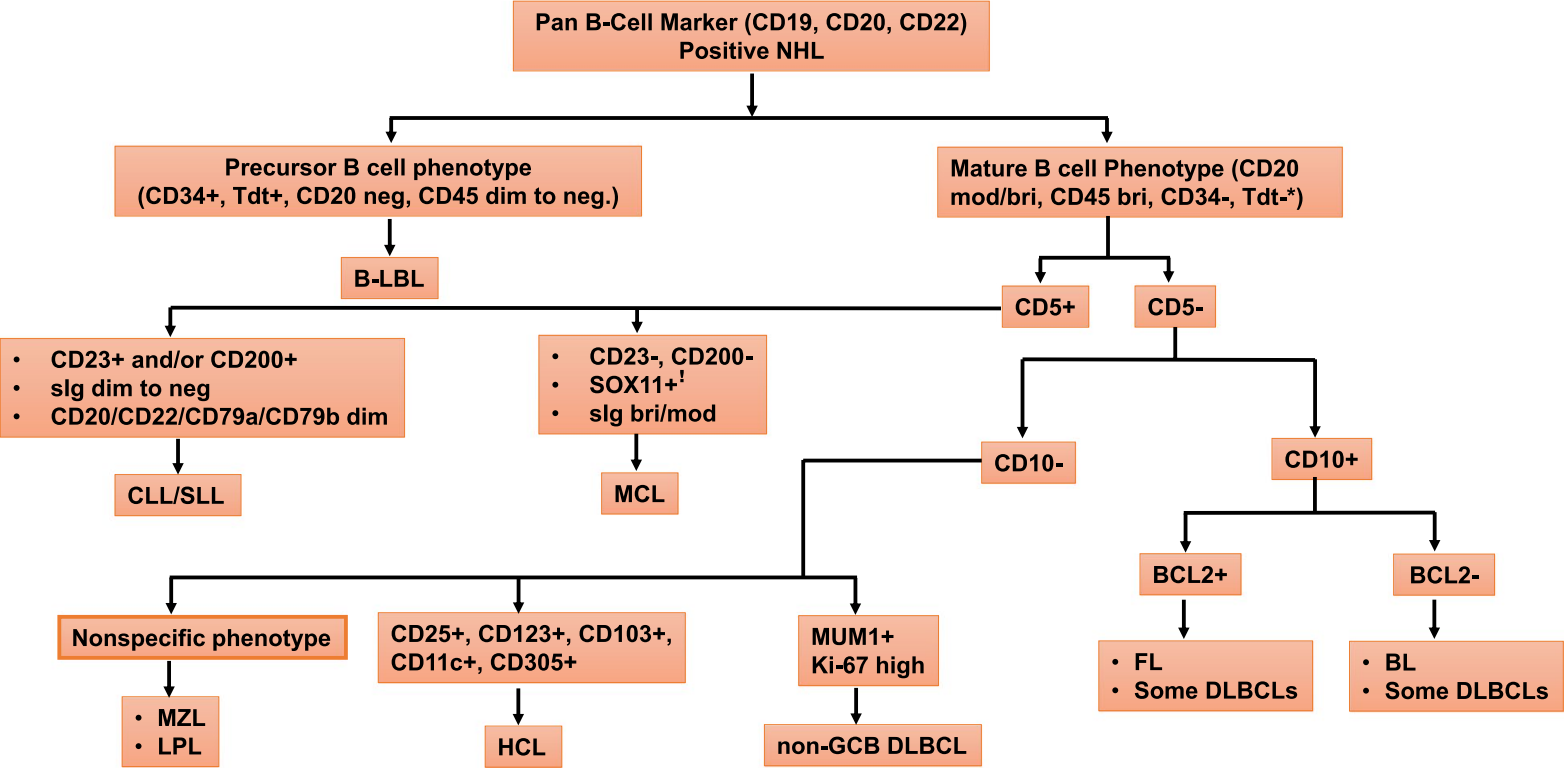
Schematic representation of immunophenotyping-based approach in mature B-cell lymphomas

1. *Ontogenetic classification —* Figure 1 shows the stages of normal B-cell ontogenetic differentiation (Fig. 1). However, the B-NHL classification based on normal B-cell ontogenesis is inadequate considering the fact that the presence of lineage heterogeneity and, more rarely, lineage plasticity in few subgroups failed to support this approach. The latest World Health Organization Blue Book emphasizes on an integrated multiparametric approach for these lymphomas [1].
2. *Morphological classification —* Categorization into small, intermediate, and large cell lymphomas based on cell size and morphological assessment is helpful; however, within the individual disease entity itself, considerable variations exist. Within MCL, blastic or pleomorphic morphology has been described, and similarly, large cells with cytologic pleomorphism are a feature of Burkitt-like lymphoma with *11q* aberration. Also, double-/triple-hit lymphomas may show intermediate to large cells or even blastic morphology [1, 15, 16]. Interobserver variation also poses a difficulty in morphologically dubious cases.
3. *Immunophenotypic classification —* Immunophenotyping and IHC are so far the most important diagnostic tools for both classification and assessment of aggressiveness of individual disease (Fig. 2). For instance, CD5+ expression in a neoplastic B-NHL delineates a differential diagnosis between small lymphocytic lymphoma (SLL)/chronic lymphocytic leukemia (CLL) vs MCL based on CD23, CD200, cyclin D1 (CCND1) and antigenic intensity of surface heavy chain, CD79a, and CD20. However, entities like atypical CLL with bright surface heavy chain expression and loss of CD23 exist and likewise cases of CD10+ MCL and leukemic non-nodal MCL with CD23 and/or CD200 expression [17–19]. Similarly, one poor prognostic subgroup of DLBCL has been described with CD5+ expression and need to be distinguished from the blastoid or pleomorphic variant of mantle cell lymphoma [1]. Distinction between LPL and marginal zone lymphoma (MZL) through immunophenotyping alone is hazy due to lack of specific antigenic markers. Double-/triple-hit lymphomas occasionally fail to demonstrate light chain restriction on immunophenotyping or IHC and may even show sparse Tdt expression creating confusion with lymphoblastic lymphomas [20].
4. *Clinical classification —* A wide range of clinical behavior is seen within any entity of B-NHL, and histological transformation as well as clinical progression (e.g., FL into DLBCL or double-hit lymphoma and SLL/CLL with Richter’s transformation) may be encountered during the course of the disease [21–23]. Therefore, stratification of these malignancies on the basis of histologic grade or clinical aggressiveness alone is unwarranted. Moreover, as a part of the clonal evolution process, lymphomas acquire additional genetic aberrancies and display both morphological and antigenic variation.

The genomic studies in this context have proven to be a crucial tool for restructuring B-NHL classification time and again. Polymerase chain reaction (PCR) of *immunoglobulin* (*IG*) gene rearrangement is a valuable technique for clonality assessment in neoplastic B cells. *CCND1* rearrangement is seen not only in classical MCLs but also in leukemic non-nodal MCLs and is virtually absent in both SLL/CLLs as well as CD5+ DLBCLs [19, 24]. Distinction between HCL and hairy cell leukemia variant (HCL-v) is based on *BRAF* mutation with the former being positive in most of the cases [10, 25]. The identification of MYD*88 L265P* and *CXCR4 S338X* mutation is of immense importance for diagnosis of LPLs with none to rare cases of MZL showing these abnormalities [11, 26, 27]. Demonstration of *MYC*, *BCL2*, and/or *BCL6* rearrangement is helpful for diagnosing HGBLs with unusual immunophenotypes or lacking light chain restriction [20, 28]. Based on gene expression profile (GEP), newer prognostic molecular models have been introduced in DLBCLs [29, 30]. Even the distinction between classical and leukemic non-nodal MCL is based on clinical features along with *SOX11* mutation study with leukemic non-nodal MCL being typically *SOX11* negative. The need for distinction between these two entities is essential as the patients with leukemic non-nodal MCL have been reported to have a longer treatment-free survival and OS compared to classical variants [31, 32]. An overview of the critical genetic aberrancies associated with major B-NHLs is highlighted in Table 1.

**Table 1:** Genetic aberrancies associated with major B-NHLs

Type of B-cell lymphoma	Affected pathway	Genes involved
Chronic lymphocytic leukemia/small lymphocytic lymphoma (CLL/SLL)	● BCR signaling ● Cell cycle and DNA damage ● RNA splicing ● Epigenetic modifiers ● NF-κB signaling ● NOTCH1 signaling ● Copy number aberrations	BCOR, CAR11, MYD88, PAX5 ATM, CDKN2A, POT1, TP53 DDX3X, SF3B1, XPO1 ARID1A, CHD2, KMT2D, SETD2 BIRC3, EGR2, TRAF2, TRAF3 NOTCH1 Del13q14.3+12
Burkitt lymphoma (BL)	● BCR signaling ● Cell cycle and DNA damage ● NF-κB signaling ● Rearrangements	ID3, TCF3 CCND3, RHOA, SMARC, TP53 TRAF1, TRAF2 c-MYC translocation t (8;14); t(8;22); t(2;8)
Follicular lymphoma (FL)	● mTOR signaling ● Tumor suppressor ● Epigenetic modifiers ● Copy number aberrations ● Rearrangements	RRAGC TNFRSF14 CREBBP, EZH2, HDAC7, KMT2D +1, +18q, +loss of 6q t (14;18) (q32; q21)
Germinal-center B-cell-like diffuse large B-cell lymphoma (GCB-DLBCL)	● Epigenetic modifiers ● PI3K/AKT signaling ● JAK/ STAT signaling ● Copy number aberrations ● Rearrangements ● Recurrent mutations	CREBBP, EZH2, KMT2D PTEN, TNFRSF14 ETV6, IL2RA, STAT3 MDM2, REL BCL2 B2M, GNA13, MEF2B, SOCS1, TP53
Activated B-cell-like diffuse large B-cell lymphoma (ABC-DLBCL)	● BCR signaling ● Cell cycle and DNA damage ● Epigenetic modifiers ● NF-κB signaling ● Copy number aberrations ● Rearrangements	CARD11, CD79A, CD79B, MYD88 CDKN2A, TP53 CREBBP, KMT2D TNFAIP3, TRAF2, TRAF5 +3q27, BCL2, PRDM1 BCL6
Mantle cell lymphoma (MCL)	● Cell cycle and DNA damage ● Copy number aberrations ● Rearrangements ● Recurrent mutations	ATM, CDKN2A, CHK2, TP53 +3q26, loss of 1p, TNFAIP3, 9p21 CCND1, CCND2, CCND3, t(11;14) CCND1, NOTCH1, SOX11
Lymphoplasmacytic lymphoma (LPL)	● NF-κB signaling ● Copy number aberrations ● Recurrent mutations	MYD88 L265P Del 6q, +4 ARID1A, CXCR4 S338X, TP53
Hairy cell leukemia (HCL)	● Cell cycle and DNA damage ● Epigenetic modifiers ● MEK-ERK signaling ● Transcription factor	CDKN1B/p27 KMT2C BRAF V600E, MAP2K1 KLF2
Primary mediastinal B-cell lymphoma (PMBL)	● NF-κB signaling ● JAK/STAT signaling ● Copy number aberrations ● Recurrent mutations	TNFAIP3 PTPN1, SOCS1, STAT6 PDL1/PDL2, REL CIITA, PDL1/PDL2

## Genetic alterations and molecular mechanisms in B-NHLs

### Small lymphocytic lymphoma/chronic lymphocytic leukemia

The B-cell receptor consisting of IG molecule and CD79A/B subunits is central to CLL pathogenesis [33]. Two major molecular subgroups have been identified based on mutational status of *immunoglobulin heavy chain variable region* (*IGHV*) genes, i.e., unmutated *IGHV* (≤ 98% germline identity) in 50–70% patients and mutated *IGHV* (≥ 98% identity) in 30–50% patients. B-cell receptor stereotype with very similar, but not identical *IG* sequences is also noted in approximately 30% of the CLL cases [34, 35]. Also, 80–90% of SLL/CLL cases have cytogenetic abnormalities detected by interphase FISH and/or copy number arrays, but disease-specific aberrancies are still unknown. The most common genetic anomalies include deletions in *13q14.3* (*miR16-1* and *miR15a*), followed by trisomy *12* or partial trisomy of *12q13* and less frequently deletion in *11q22-23* (*ATM* and *BIRC3*), *17p13* (*TP53*), or *6q21* [1]. Gene mutations are also seen in 3–15% of CLL cases most commonly involving *NOTCH1*, *SF3B1*, *TP53*, *ATM*, *BIRC3*, *POT1*, and *MYD88* genes [36, 37]. Mutated *NOTCH1*, *ATM*, *SF3B1*, and *TP53* are typically associated with a shorter OS and progression-free survival in treatment-naïve CLL patients [38]. Altered expression patterns of other regulatory microRNAs apart from *miR16-1* and *miR15a* also play a dynamic role in CLL development and progression. Differential expression of *miR-34a*, *miR-223*, *miR-150*, *miR-181*, and *miR-33b* has been observed in *17p13* deleted subgroups. Moreover, *miR-4524a* and *miR-744* have shown to be associated with shorter time to first treatment [39, 40].

DNA methylation plays variable functional roles linked to CLL pathogenesis and disease outcome [41]. One study proposed a classification of CLL cases into three groups, based on their DNA methylation profiles, i.e., naive B-cell-like, memory B-cell-like, and intermediate CLL. Prognostically, memory-like cases have the best outcome followed by intermediate, whereas naive-like cases are associated with an unfavorable prognosis [42]. Few signature epigenomic events include DNA hypomethylation-induced upregulation of *TP63*, *ZAP70*, and *NFATc1* and downregulation of *DUSP22* and *KLF4* responsible for aggressive course of disease [43–46]. Also, upregulated expression of *PAX9* and *CRY1* has been shown to have a shorter treatment initiation interval, and *PAX9* particularly is associated with a shorter OS [47]. Recent epigenetic models point towards an aging-related increase in DNA methylation at specific genomic regions, particularly single *CpG* sites that give rise to different clones with divergent outcomes. One such example is a single non-promoter *CpG+223* methylation critical for *ZAP70* expression [48].

### Mantle cell lymphoma

The crucial molecular mechanism in MCL lies in the overexpression of *CCND1* molecule in the naive pre-germinal-center B cells. Aberrant truncated mRNA transcripts of *CCND1* are particularly associated with aggressive course and poor prognosis. Majority of MCL cases are associated with *t(11;14) (q13;q32)* translocation between the *IGH* gene and *CCND1* [24]. *Cyclin D1/CDK4/6* complex-induced phosphorylation and subsequent inactivation of *RB* gene lead to progression of cells from G1 to the S phase resulting in rapid cell proliferation. One key event in this process is the perinucleolar repositioning of the rearranged *IgH-CCND1* segment in nucleolin transcription factor-rich areas [49–51]. In addition, abnormal overexpression of *SOX11* is specific for MCLs independent of *t(11;14)*. In *CCND1*-negative patients, *SOX11* positivity as well as *cyclin D2* (*CCND2*)/*cyclin D3* (*CCND3*) translocations is of diagnostic utility, and such patients behave identically to that of CCND1-positive ones [52, 53]. Additional alterations in genes targeting cell cycle regulatory elements, the DNA damage response pathway, and cell survival most notably *ATM*, *KMT2D*, *NOTCH1/2*, *NSD2*, *BIRC3*, *TRAF2*, *MAP2K14*, *CARD11*, *SMARCA4*, *UBR5*, and *BTK* have been demonstrated in various morphological subgroups. Moreover, TP53 mutations in particular are associated with progression to blastoid or pleomorphic MCL [54–56]. Leukemic non-nodal variant of MCL is also associated with *CCND1* translocation; however, *SOX11* mutation has never been reported [31, 32].

### Follicular lymphoma and variants

Follicular lymphoma is a heterogeneous neoplasm, and the latest WHO classification describes different clinicobiological variants, namely nodal FL, in situ follicular neoplasia (ISFN), duodenal-type FL, testicular FL, diffuse FL, and pediatric type. Through multiplex PCR, somatic hypermutation of *IGH VDJ* gene through acquisition of asparagine (N)-linked glycosylation sites is detected in almost all the cases of FL [57, 58]. The aberrant genetic hallmark characterized by the *t(14;18) (q32;q21)* is identified in as many as 90% of grades 1–2 nodal FLs and involves translocation between the *IGH* and *BCL2* genes resulting in the antiapoptotic protein BCL2 overexpression [57]. Nevertheless, BCL2 translocation is generally absent in testicular, diffuse, and in pediatric variants [1, 59, 60]. Additional genetic alterations in nodal FL include loss of *1p*, *6q*, *10q*, and *17p* and gains of chromosomes *1*, *6p*, *7*, *8*, *12q*, *X*, and *18q* [1]. Specifically, in ISFN, comparative genomic hybridization array studies have identified low amplitude copy number amplifications of chromosomes *1* and *18* which could be an early step in lymphomagenesis. Apart from the driver oncogenes, few recently discovered genes particularly related to tumor biology in ISFN include *BACH2*, *TOX*, *AFF3*, and *EBF1* [61]. One noteworthy mutation in chromosome *1p36* region involving *TNFRSF14* gene is present in all FLs including the diffuse and pediatric-type variant. Moreover, somatic mutations *RRAGC* gene locus at *1p34.3* resulting in activated *mTORC1* downstream pathway are found in approximately 17% of cases [62, 63]. Gene expression profiling also recognizes specific mutations within individual variants of FL. Particularly in pediatric-type FLs, *MAP2K1* gene encoding the MEK1 protein is the most commonly mutated gene followed by *TNFRSF14* [64]. Similarly, duodenal-type FLs typically overexpress *CCL20* and *MADCAM1* genes resembling the mutational profile of MALT lymphoma [65].

Epigenetic aberrancy in FLs tumorigenesis is a well-established phenomenon. Amplifications of histone modifiers *EZH2*, *ARID2*, and *HDAC7* as well as chromatin regulators *CREBBP* and *KMT2D* are commonly noted in FLs and appear to be an early driving event [61, 66]. However, one exception to this finding is pediatric-type FL which usually lacks recurrent mutations of epigenetic modifiers [58, 61, 64].

### Lymphoplasmacytic lymphoma

A diagnostic challenge remains to differentiate between LPL and marginal zone lymphoma on the basis of morphology and immunophenotype. However, with the detection of *MYD88 L265P* mutation in > 90% cases of LPLs, this distinction is now easier. *MYD88 L265P* mutation activates the *NF-κB* pathway through phosphorylation of *Bruton’s tyrosine kinase* (*BTK*) in the B-cell receptor [26, 27, 67]. In addition, approximately 30% LPL patients have truncating *CXCR4* mutations most frequently *S338X* (nonsense), followed by *S341fs* (frameshift) and *R334X* (nonsense) mutation [27]. Other somatic mutations, such as mutations of *ARID1A*, *TP53*, *CD79B*, *KMT2D*, and *MYBBP1A*, are encountered with lower frequency. Mutations and deletion of *TP53* are typically associated with an unfavorable prognosis. *6q deletion* and *trisomy 4* are the most common cytogenetic abnormalities demonstrated in 7–54% and 20% of patients with Waldenstrom macroglobulinemia (WM) respectively which can be of additional diagnostic utility [1, 26, 27].

### Hairy cell leukemia and hairy cell leukemia variant

The high frequency of *BRAF V600E* mutation, described by multiple studies, plays a key role in the pathogenesis of HCL. The mutation is located at *exon 15* of *BRAF* gene on chromosome *7q34* leading to valine substitution by glutamate at codon 600 (*V600E*) of the *BRAF* protein. This promotes autophosphorylation of *BRAF* protein and constitutive activation downstream *MEK-ERK* signaling pathway [10, 25]. *BRAF* mutation is reported to be absent in 10 to 20% of patients with HCL. However, such patients may demonstrate mutations at alternate exon sites such as exon 11. In *BRAF* wild-type patients, mutations in *MAP2K1* gene encoding *MEK* are commonly encountered [68].

Apart from these signature aberrancies, few cases show recurrent loss-of-function mutation in *CDKN1B/p27* and *KLF2*; however, *TP53* mutations or *del17p* are rare. Among the epigenetic regulators, mutations in the histone methyltransferase *KMT2C* are found in approximately 15% of patients [69, 70].

In contrast to HCL, mutations of *BRAF* are not seen in HCL-v, but activating mutations in *MAP2K1* are detected in half of the cases. Other less commonly identified mutations are noted in cell cycle regulator *CCND3* and spliceosome encoding gene *U2AF1*. Compared to HCL, HCL-V shows more frequent recurrent mutations in epigenetic modifiers *KMT2C*, *CREBBP*, *KDM6A*, and *ARID1A* [69–71].

### Burkitt lymphoma and Burkitt-like lymphoma with 11q aberration

Burkitt lymphoma is a highly aggressive lymphoma, and the key oncogenic event is the translocation between *c-MYC* gene at band *8q24* and *IGH* region on chromosome *14q32 [t(8;14) (q24;q32)]* or less commonly to the *IGK* gene locus at *2p12 [t(2;8)]* or the *IGL* locus at *22q11 [t(8;22)]*. Epstein-Barr virus, an established causative pathogen, can be identified in all cases of endemic BL and approximately 20 to 40% of cases of sporadic and immunodeficiency-associated BL through Epstein-Barr encoding region (*EBER*) in situ hybridization [72, 73]. In approximately 70% of sporadic BL cases, factor *TCF3* or its negative regulator *ID3* mutation is demonstrated by whole-genome or transcriptome sequencing. These mutations activate antigen-independent B-cell receptor signaling through the Pl3K pathway which promotes BL cell survival [73, 74]. Other less commonly encountered genes involved in BL tumorigenesis include *CCND3*, *TP53*, *RHOA*, *SMARCA4*, *ARID1A*, *IGLL5*, *BACH2*, *SIN3A*, and *DNMT1*. Immunodeficiency-associated BL share an identical genetic profile found in sporadic type. However, endemic BL often harbors mutations in *BCL7A* as well as *BCL6* and less commonly alterations in *DNMT1*, *CCND3*, *ARID1A*, and *RHOA* genes. The incidence of *TCF3* or *ID3* driver mutations is also lower than the sporadic type [74]. Moreover, individuals with *SH2D1A* gene-mutated X-linked lymphoproliferative syndrome have shown to be highly susceptible for developing BL [75].

The latest WHO classification has subdivided the previous “Burkitt-like” lymphoma category into more precisely defined entities like Burkitt-like lymphoma with *11q* aberration, high-grade B-cell lymphoma with *MYC* and *BCL2* and/or *BCL6* rearrangement, and high-grade B-cell lymphoma, not otherwise specified.

Burkitt-like lymphoma with *11q* aberration is a new entity with a complex karyotype and lacking *MYC* rearrangement or *1q* gain typical of BL. These lymphomas often display mild to moderate cytologic pleomorphism and a nodal presentation, predominantly with a single bulky tumor mass. The alteration of chromosome *11q* is characterized by interstitial amplification in *11q23.2-23.3* region and telomeric losses of *11q24.1-qter* [76, 77]. Studies have demonstrated that these lymphomas have high-grade gene expression profiling, and their mutational profile resembles that of germinal center-derived lymphomas (also supported by *LMO2* expression). Most commonly occurring recurrent gene mutations include *BTG2*, *DDX3X*, and *ETS1*; however, mutations in *ID3*, *TCF3*, or *CCND3* genes typical of BL are primarily absent [77, 78]. A significant association has been demonstrated with mutations in epigenetic modifier genes such as *CREBBP*, *KMT2C*, *ARID1A*, *EP300*, *CREBBP*, *KMT2C*, *EZH2*, and *KMT2D*; however, the data is limited owing to the rarity of the disease [78].

### Diffuse large B-cell lymphoma

Diffuse large B-cell lymphoma is the most commonly occurring NHL with a high refractoriness and relapse rate. Molecular heterogeneity is a hallmark of DLBCL, and several studies have demonstrated a diverse gene expression profile. In the cell-of-origin classification, transcriptional subtypes of DLBCL encompassing activated B-cell like (ABC) and germinal center B-cell like (GCB), and molecular high-grade variants, have been described [79]. Recurrent somatic gain-of-function mutations in histone-lysine N-methyltransferase *EZH2* as well as loss-of-function mutations *GNA13* genes are fundamental molecular aberrancies demonstrated in GCB DLBCL [1, 80]. Similarly, 40% cases of GCB DLBCL show translocation of the *BCL2* gene, a characteristic of FL. In contrast, ABC DLBCLs frequently demonstrate mutations of genes linked to *BCR* signaling and *NF-κB* pathways, i.e., *CD79A*, *CD79B*, *CARD11*, *MYD88*, *TNFAIP3*, *TRAF2*, and *TRAF5*. Translocation of the 3q27 region involving *BCL6* gene tends to occur more commonly in ABC type as well [81].

Apart from these genetic aberrancies, GCB DLBCL express copy number alterations in terms of amplifications of *REL*, *MDM2*, and *MIRHG1* genes as well as deletions in *TNFRSF14*, *PTEN*, and *ING1*. Loss of *PTEN* particularly provides an oncogenic signal through constitutive activation of the PI3K*/AKT* pathway. At the same time, ABC DLBCL cases show gain of *FOXP1*, *NFKBIZ*, *BCL2*, and *NFATc1* and deletion of *PRDM1* as well as *INK4/ARF* locus encoding *CDKN2A* tumor suppressor gene [82, 83].

A recent study has classified DLBCLs through targeted sequencing into five molecular subgroups based on their genomic profile and different outcomes, i.e., MYD88, BCL2, SOCS1/SGK1, TET2/SGK1, and NOTCH2 types [29]. The MYD88 subgroup comprises mutations in *MYD88* L265P, *PIM1*, *CD79B*, and *ETV6*, and loss of *CDKN2A* belongs to the ABC type and is associated with a poor prognosis. BCL2 type demonstrates recurrent mutations of *EZH2*, *BCL2*, *CREBBP*, *TNFRSF14*, *KMT2D*, and *MEF2B*, belongs to GCB type, and generally has a favorable prognosis. The most favorable outcome is associated with SOCS1/SGK1 subgroup harboring mutations of *SOCS1*, *CD83*, *SGK1*, *NFKBIA*, and *HIST1H1E* and predominantly of GCB type. TET2/SGK1 is a less commonly delineated subtype having a similar genomic profile as that of SOCS1/SGK1 but lacks *SOCS1* as well as *CD83* mutations and instead carries additional mutations of *TET2* and *BRAF*. This too is predominantly of GCB origin and shows a favorable prognosis. The 5th subgroup, i.e., NOTCH2 type dominated by mutations of *NOTCH2*, *BCL10*, *TNFAIP3*, *CCND3*, and *SPEN*, is not associated with any cell of origin and in fact resembles the tumor biology of MZLs. Another study too suggested a novel molecular classification for DLBCLs and categorized them into five distinctive genetic clusters (C1–C5) [30]. A detailed description of individual subtypes is beyond the scope of this article. Roughly, the MYD88 cluster strongly recapitulates with the C5 subgroup, BCL2 with C3, TET2/SGK1 with C4, and NOTCH2 with that of C1. The distinct C2 subtype shows biallelic *TP53* inactivation, loss of *CDKN2A*, and widespread copy number changes and carries a dismal prognosis.

Moreover, DLBCLs predominantly arising in testis and central nervous system demonstrate immune escape phenomenon through downregulation of the MHC class II transactivator *CIITA* which encodes for HLA I and HLA II and thereby leading to MHC II silencing [84]. Loss of function of *beta2-microglobulin* gene, a component of HLA heavy chain, is also noted in one-third of DLBCL cases facilitating immune escape [85].

### Primary mediastinal (thymic) large B-cell lymphoma

Primary mediastinal (thymic) large B-cell lymphoma (PMBL) is an aggressive mature B-cell NHL with unique clinicopathologic features. The key biological aberrancy is mutations or rearrangements of transactivator *CIITA* gene at chromosome *16p13* leading to MHC class II molecule downregulation [84, 86]. Most common partner genes for rearrangement of *CIITA* are *programmed cell death ligands 1* and *2* (*PDL1* and *PDL2*). *REL* and *BCL11A* gene amplifications are identified in around 50% cases of PMBL. Constitutive activation of *NF-κB* and *JAK/STAT* pathways is essential for PMBL pathogenesis [87]. Recent studies suggest that deletion of *TNFAIP3* tumor suppressor gene in turn promotes *NF-κB* signaling in approximately 60% patients [88]. On the other hand, amplification of the subtelomeric region of chromosome *9p24.1* together with loss-of-function mutation of *SOCS1* and *PTPN1* activates the *JAK/STAT* pathway [87, 89]. However, rearrangements of *MYC*, *BCL2*, and *BCL6* are generally not reported in PMBL.

### Large B-cell lymphoma with IRF4 rearrangement

Large B-cell lymphoma (LBCL) with *IRF4* rearrangement is a rare subtype of LBCL with a favorable outcome predominantly occurring in pediatric and young adolescents. It often involves the Waldeyer’s ring or head and neck lymph node and shows a characteristic cryptic rearrangement of the IRF gene at chromosome *6p25* locus [90, 91]. The partner gene is most often IGH, and light chains are involved rarely. In situ hybridization using a break-apart probe for *IRF4* is a simple and easy technique for diagnosing such cases [1, 91]. Recent studies have identified frequent mutations in *IRF4*, *CARD11*, *CD79B*, and *MYD88* genes and possible activation of *NF-κB* pathway responsible for tumorigenesis. Almost all cases lack *MYC* or *BCL2* rearrangement, whereas *BCL6* breakpoints may be detected occasionally [92, 93].

### High -grade B-cell lymphoma with MYC and BCL2 and/or BCL6 rearrangements

High-grade B-cell lymphoma with *MYC* and *BCL2* and/or *BCL6* rearrangements is an aggressive form of B-cell lymphoma carrying a *MYC* rearrangement at chromosome *8q24* accompanied by additional translocations of *BCL2* at chromosome *18q21* and/or *BCL6* at chromosome *3q27*, also known as double-hit or triple-hit lymphoma [28, 94]. In approximately 65% of cases, the partner gene for *MYC* is one of the *IG* genes frequently *IGH* followed by *IGK* or *IGL* [94]. These lymphomas are often associated with a complex karyotype and may demonstrate hemizygous mutations of *ID3* [1, 95]. Recent studies suggested that double-hit lymphomas have an identical mutation profile (recurrent mutations in *BCL2*, *KMT2D*, *CREBBP*, *EZH2*, and *TNFRSF14*) seen in FLs, and therefore, the possible origin might be FLs or its precursor lesion. It also emphasizes the fact that additional mutations in *MYC*, *TP53*, *GNA13*, *P2RY8*, *PIM1*, and *B2M* genes displayed by FLs during high-grade transformation are detected in double-hit HGBLs as well [96, 97].

## Overlapping molecular aberrancies in B-NHLs

More than 20 lymphoproliferative disorders are classified under B-cell NHLs. Nonetheless, these share considerable resemblance not only in terms of morphology and immunophenotype alone but genomic profile as well. The hallmark *MYD88 L265P* driver mutation for LPLs is also detected in CLLs, MZLs, MCLs, ABC DLBCLs, and primary CNS lymphomas with a much lower frequency [98]. It should be emphasized here that the cell of origin for all these lymphomas is not the same. Similarly, *c-MYC* rearrangements tend to occur in indolent low-grade B-cell lymphoma as well as highly aggressive BL, plasmablastic, and double-hit lymphomas [99]. At the same time, some GCB DLBCLs also demonstrate *t(14;18)* observed in FLs as already mentioned above, and in this case, both of these lymphomas originate from germinal center B cells. Moreover, CD30+ DLBCLs show an overlapping gene expression profile with that of PMBL. Similar patterns of mutation in *KMT2C*, *TP53*, and *LAMA3* have been described in BLs and DLBCLs. Loss-of-function mutation of *TNFAIP3* is found in ABC DLBCLs, PMBLs, classical Hodgkin lymphomas, and MCLs [88]. Also, the acquisition of additional hallmark genetic abnormalities during high-grade transformation of indolent lymphomas is a well-documented phenomenon. For example, CLL transformation to DLBCL shows a number of acquired anomalies including *NOTCH1* and *TP53* mutations, *MYC* translocations, and loss of function of *CDKN2A* [100, 101]. Likewise, FLs may progress to double-hit lymphomas with additional rearrangements of *BCL2* and *MYC*. Another aspect is that neither all cases of a specific lymphoma express its hallmark genetic anomaly nor the gene expression profile is the same for each case. These molecular features are very similar to the immunophenotyping findings observed in these lymphomas.

## Molecular alterations in tumor microenvironment

**Fig. 3:**
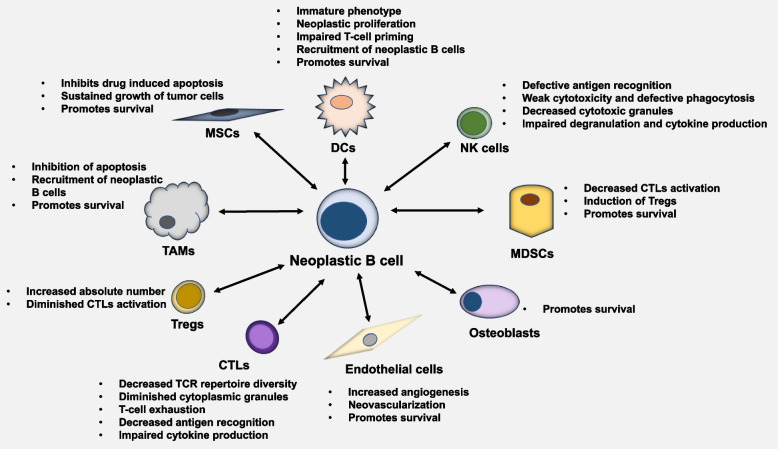
Interaction with components of tumor microenvironment and immune-driven mechanisms in B-cell lymphomas

The tumor microenvironment (TME) in B-NHLs is heterogeneous and composed of the cellular compartment of immune and inflammatory cells, fibroblasts, endothelial cells, and lymphovascular networks and the extracellular matrix [102]. Lymphoma cells are dependent on TME for the regulation of tumor cell survival. Figure 3 demonstrates the detailed interaction of various TME cells with tumor cells in B-NHLs. Nonetheless, the interaction between TME and lymphoma cells plays a critical role in the lymphomagenesis in one of the following ways: (1) escape of lymphoma cells from immune surveillance and (2) emergence of drug resistance. Immune escape mechanism is a well-recognized phenomenon in both DLBCLs and PMBLs. The MHC class II transactivator *CIITA* is commonly mutated in DLBCLs and PMBLs resulting in T-cell exhaustion [87]. In addition, copy number gains of *PDL1* and *PDL2* appear to regulate immune escape in PMBLs. *PDL1* overexpression on tumor-associated macrophages has been linked to adverse prognostic subgroups in DLBCLs. The same *PDL1* gene mutation in MCL results in dysregulated T-cell proliferation and impaired antitumor response [103, 104]. *B2M* and *CD58* genes are critical for recognition of tumor antigen by circulating T lymphocytes and NK cells, respectively. Loss-of-function mutation or deletion of *B2M* and *CD58* genes is responsible for evading this immune cell recognition in cases of DLBCL [85]. Also, there is an increased VEGF expression and higher microvascular density found in CD5+ABC DLBCLs than GCB DLBCLs [105, 106]. This finding is in accordance with increased pro-angiogenic microRNAs expression, i.e., *miR-126* and *miR-130a*, in both DLBCL tumor cells and TME cells [107]. In FLs too, GEP of tumor-infiltrating lymphocytes (TILs) has shown that patients with ≤ 5% PD1+ TILs are more likely to progress to high-grade lymphomas [108].

For DLBCL, an international prognostic system (IPS) incorporating simple clinical parameters (age, lactate dehydrogenase, number/sites of involvement, stage, performance status) is widely used. However, IPI is inadequate to identify patients with poor survival; thus, integration of molecular and genetic features of the tumor and its microenvironment into existing scoring systems is strongly recommended for better risk stratification. In this context, a risk model combining clinical parameters of the IPI with genetic aberrancies is (KMT2D, PIM1, and MEF2B) is proposed which identifies patients with a poorer prognosis than the highest-risk IPI category, but it has not been externally validated in a large group of patients [109].

## Role of genetic aberrancies in drug resistance in B-NHLs

**Fig. 4:**
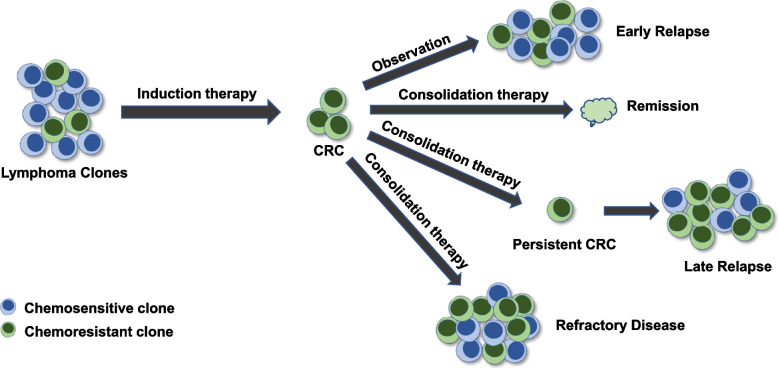
Drug-resistant subclones modulating relapse and refractory disease course in B-cell malignancies

Molecular aberrancies and genomic instability in tumor cells leading to emergence of heterogeneous subclones underlie the primary mechanism of chemoresistance in B-NHLs (Fig. 4). *BTK*, a key intermediate of *BCR* signaling and NF-κB anti-apoptotic pathways, is widely targeted in lymphomas by ibrutinib, a novel BTK inhibitor. The *C481S* mutation in *BTK* gene as well as mutation in *Pim-1 proto-oncogene* (*PIM1*), a serine/threonine kinase, led to ibrutinib resistance in ABC DLBCLs [110, 111]. Similarly, concurrent mutation in *CXCR4 S338X* driving the PI3K/AKT and ERK pathways is responsible for acquired ibrutinib resistance in one-third of *MYD88 L265P*-mutated WM patients. In contrast, the presence of wild-type *MYD88* in WM patients displays similar drug resistance and shorter progression-free survival as well as OS [112, 113].

In DLBCL patients, mutations of genes regulating *BCR* signaling pathway especially *CD79A/B* and *CARD11* show refractory disease course [114]. In ibrutinib-treated MCL patients, mutations of *TP53* and *NSD2* as well as loss of function of *PTEN* and *FOXO3a* have shown to be associated with blastoid transformation [115]. One noteworthy example is gain-of-function rearrangement of *BMI-1* gene at chromosome *10p12* that leads to development of drug-resistant phenotype through transcriptional repression of pro-apoptotic genes in indolent lymphomas like CLLs, FLs, and MCLs. Loss-of-function mutation of *CDKN2A* was shown to be responsible for chemoresistance in MCLs and DLBCLs. Also, in CLL/SLL patients, mutation or deletion in *BIRC3* and *SF3B1* genes has been associated with fludarabine resistance [116]. IL6-induced *JAK/STAT* signaling has been linked to acquired resistance to *PI3K* pathway inhibitors copanlisib and duvelisib [117]. Similarly, one hypothesis suggests a hyperactivated insulin-like growth factor 1 receptor-related mechanism for idelalisib refractoriness in CLL patients with trisomy *12* [118, 119].

It is widely accepted that *BCL2* anti-apoptotic protein overexpression either through *t(14;18)* or silencing of microRNAs *miR15a* and *miR16-1* is associated with inherent as well as acquired chemoresistant subclones within B-NHLs [119]. In addition, there is increasing evidence to class I BCL2 inhibitor venetoclax recently in MCL and CLL patients which has been related to mutation in *BH3* drug binding domain of *BCL2* gene, loss of *BIM*, or overexpression of *MCL-1* and *BCL-XL* genes [120, 121]. In the era of targeted therapies, a deeper understanding of all these molecular mechanisms regarding drug resistance will indeed help in optimizing treatment protocols.

## Conclusion

In conclusion, the spectrum of molecular and genomic landscapes of B-NHLs is ever evolving. Considerable genetic variations and overlapping molecular traits observed across the various subgroups are highlighting the role of emerging techniques, viz., molecular analysis at single-cell level and proteomics in precise diagnosis, classification, and risk stratification of B-NHLs. Single-cell sequencing may open new facets of molecular mechanisms which may prove to be a key element for translational research. Furthermore, with the increasing evidence of the potential role of tumor microenvironment on lymphomagenesis as well as in drug resistance, integration of the genomic landscape with microenvironment composition is essential to get better insight of the therapeutic modalities of B-NHL improving the overall treatment outcome.

## Data Availability

Not applicable

## Abbreviations

ABC: Activated B-cell like
BCR: B-cell antigen receptor
BLs: Burkitt lymphomas
B-NHLs: B-cell non-Hodgkin lymphomas
BTK: Bruton’s tyrosine kinase
CCND1: Cyclin D1
CCND2: Cyclin D2
CCND3: Cyclin D3
CLL: Chronic lymphocytic leukemia
DLBCLs: Diffuse large B-cell lymphomas
EBER: Epstein-Barr encoding region
FISH: Fluorescence in situ hybridization
FLs: Follicular lymphomas
GCB: Germinal center B-cell like
GEP: Gene expression profile
HCL: Hairy cell leukemia
HCL-v: Hairy cell leukemia variant
HGBLs: High-grade B-cell lymphoma
IG: Immunoglobulin
IGHV: Immunoglobulin heavy chain variable region
IHC: Immunohistochemistry
ISFN: In situ follicular neoplasia
LBCL: Large B-cell lymphoma
LPL: Lymphoplasmacytic lymphoma
MCLs: Mantle cell lymphomas
MLPA: Multiplex ligation-dependent probe amplification
MZL: Marginal zone lymphoma
NF-κB: Nuclear factor kappa B
OS: Overall survival
PCR: Polymerase chain reaction
PDL1: Programmed cell death ligand 1
PDL2: Programmed cell death ligand 2
PMBL: Primary mediastinal (thymic) large B-cell lymphoma
SLL: Small lymphocytic lymphoma
TILs: Tumor-infiltrating lymphocytes
TME: Tumor microenvironment
WM: Waldenstrom macroglobulinemia
